# *De novo* Biosynthesis of Odd-Chain Fatty Acids in *Yarrowia lipolytica* Enabled by Modular Pathway Engineering

**DOI:** 10.3389/fbioe.2019.00484

**Published:** 2020-01-22

**Authors:** Young-kyoung Park, Rodrigo Ledesma-Amaro, Jean-Marc Nicaud

**Affiliations:** ^1^Université Paris-Saclay, INRAE, AgroParisTech, Micalis Institute, Jouy-en-Josas, France; ^2^Imperial College Centre for Synthetic Biology and Department of Bioengineering, Imperial College London, London, United Kingdom

**Keywords:** odd-chain fatty acids, propionyl-CoA, *Yarrowia lipolytica*, metabolic engineering, Golden Gate assembly, synthetic biology

## Abstract

Microbial oils are regarded as promising alternatives to fossil fuels as concerns over environmental issues and energy production systems continue to mount. Odd-chain fatty acids (FAs) are a type of valuable lipid with various applications: they can serve as biomarkers, intermediates in the production of flavor and fragrance compounds, fuels, and plasticizers. Microorganisms naturally produce FAs, but such FAs are primarily even-chain; only negligible amounts of odd-chain FAs are generated. As a result, studies using microorganisms to produce odd-chain FAs have had limited success. Here, our objective was to biosynthesize odd-chain FAs *de novo* in *Yarrowia lipolytica* using inexpensive carbon sources, namely glucose, without any propionate supplementation. To achieve this goal, we constructed a modular metabolic pathway containing seven genes. In the engineered strain expressing this pathway, the percentage of odd-chain FAs out of total FAs was higher than in the control strain (3.86 vs. 0.84%). When this pathway was transferred into an obese strain, which had been engineered to accumulate large amounts of lipids, odd-chain fatty acid production was 7.2 times greater than in the control (0.05 vs. 0.36 g/L). This study shows that metabolic engineering research is making progress toward obtaining efficient cell factories that produce odd-chain FAs.

## Introduction

Microbial oils (lipids and fatty acid-derived products) are regarded as promising alternatives to fossil fuels in the face of growing concerns over environmental issues and energy production. To lessen the cost of producing microbial oils, considerable effort has been dedicated to enhancing production yield (Dulermo and Nicaud, 2011; Tai and Stephanopoulos, 2013; Qiao et al., 2015); using low-cost carbon substrates (Papanikolaou et al., 2013; Lazar et al., 2014; Guo et al., 2015; Ledesma-Amaro and Nicaud, 2016); and targeting high-value lipids (Xue et al., 2013; Xie et al., 2015). Odd-chain fatty acids (FAs), a type of valuable lipid, are a product with potential because they can be used in a variety of applications. Notably, research has revealed that odd-chain FAs with chain lengths of 15 and 17 carbons may have functional importance for nutrition and medical field. For example, *cis*-9-heptadecenoic acid has anti-inflammatory effects and can help treat psoriasis, allergies, and autoimmune diseases (Degwert et al., 1998). Pentadecanoic acid and heptadecanoic acid can be used as biomarkers of food intake in dietary assessments, the risk of coronary heart disease (CHD), and the risk of type II diabetes mellitus (Forouhi et al., 2014; Jenkins et al., 2015; Pedersen et al., 2016; Pfeuffer and Jaudszus, 2016). The chemical properties and potential biological activities of odd-chain FAs are now being more extensively studied (Rezanka and Sigler, 2009), so it is possible that novel nutritional and pharmaceutical applications could soon be discovered. In addition, odd-chain FAs and their derivatives are precursors for manufacturing substances such as pesticides, flavor and fragrance compounds, hydraulic fluids, plasticizers, coatings, and other industrial chemicals (Fitton and Goa, 1991; Avis, 2000; Clausen et al., 2010; Köckritz et al., 2010). Despite the broad range of applications for FAs, studies aiming to produce odd-chain FAs using microorganisms have had limited success because microorganisms produce a much greater proportion of even-chain FAs than odd-chain FAs.

**Figure 1:**
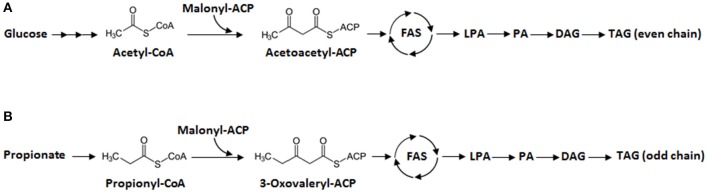
Lipid synthesis in *Y. lipolytica*. **(A)** Synthesis of even-chain fatty acids (FAs) from glucose. **(B)** Synthesis of odd-chain FAs from propionate. First, there is elongation of fatty acyl-CoA by fatty acid synthase (FAS). Second, the resulting even- or odd-chain fatty acyl-CoA is transformed into lysophosphatidic acid (LPA), phosphatidic acid (PA), and diacylglycerol (DAG), in that order, before finally forming triacylglycerol (TAG).

Generally, *de novo* fatty acid synthesis in microorganisms begins with the condensation of acetyl-CoA and malonyl-CoA (Figure 1A). Then, the elongation step occurs: long-chain FAs are synthesized in a reaction catalyzed by fatty acid synthase (FAS). The resulting acyl-CoA products are esterified to generate lysophosphatidic acid (LPA), then phosphatidic acid (PA), and finally diacylglycerol (DAG) before forming triacylglycerol (TAG), the compound in which the lipids are stored. For odd-chain FAs, propionyl-CoA could be converted from propionate is a primer for the fatty acid synthesis. The condensation of both propionyl-CoA and malonyl-CoA results in the formation of 3-oxovaleryl-ACP, which is the launching point for odd-chain FA synthesis. This five-carbon compound goes through elongation, where two carbons are added in each cycle. Then odd-chain FAs can be synthesized as described in Figure 1B. However, most microorganisms require the presence of propionate in the medium to produce odd-chain FAs. Wu and San have shown that *E. coli* can produce odd-chain FAs—namely undecanoic acid (C11:0), tridecanoic acid (C13:0), and pentadecanoic acid (C15:0)—if grown with propionate-supplemented medium (Wu and San, 2014). They introduced a propionyl-CoA synthetase (*prpE*) from *Salmonella enterica* and acyl-ACP thioesterases (TEs) from *Umbellularia californica* and *Ricinus communis*. Additionally, propionate supplementation has allowed various yeasts (e.g., *Yarrowia lipolytica, Rhodotorula glutinis, Cryptococcus curvatus*, and *Kluyveromyces polysporus*) to produce odd-chain FAs (Zheng et al., 2012; Kolouchová et al., 2015). More recently, in *Y. lipolytica*, metabolic engineering and the optimization of propionate feeding helped boost the production of odd-chain FAs, namely heptadecenoic acid (C17:1) (Park et al., 2018).

Therefore, to date, most studies have been focused on processes that involve propionate supplementation. However, due to the high cost (Poirier et al., 1995; Aldor et al., 2002) and toxic effects of propionate (Fontanille et al., 2012; Park and Nicaud, 2019), it is crucial to find alternative pathways for generating propionyl-CoA to be able to produce odd-chain FAs on large scales. There have been a few studies in which odd-chain FAs have been produced using glucose. Tseng and Prather have shown that, in *E. coli*, the production of very short odd-chain FAs (i.e., propionate, trans-2-pentenoate, and valerate) can be increased through the upregulation of threonine biosynthesis (Tseng and Prather, 2012). Another study demonstrated that the overexpression of threonine biosynthesis in *E. coli* resulted in increased levels of odd-chain FAs (mainly C15:0): from 0.006 to 0.246 g/L. This study further modified the experimental strain by replacing the native β-ketoacyl-ACP synthase (encoded by *FabH*) with one from *Bacillus subtilis* (encoded by *FabHI*) so that there was a biochemical preference for propionyl-CoA over acetyl-CoA (Lee et al., 2013). However, to date, no one has reported the *de novo* production of odd-chain FAs in *Y. lipolytica* or in any other yeasts without propionate supplementation. Consequently, we need more research on metabolic engineering approaches for producing odd-chain FAs in this group of microorganisms.

Of the several microbial hosts used in such systems, the oleaginous yeast *Y*. *lipolytica* is the most studied and has been engineered to produce large amounts of lipids and lipid derivatives, such as ricinoleic acid (Beopoulos et al., 2014), conjugated linoleic acids (Imatoukene et al., 2017), cyclopropane FAs (Czerwiec et al., 2019), and cocoa butter-like oils (Papanikolaou et al., 2003). *Y. lipolytica* can naturally grow in a broad range of substrates and has been further engineered to use even more substrates (Ledesma-Amaro et al., 2016). In addition, many different synthetic biology tools have been created for and applied in *Y. lipolytica*, such as Gibson assembly, Golden Gate assembly, TALEN editing, and CRISPR/Cas9 editing [please see the recent review by Larroude et al. (2018) for more details]. These tools used in tandem with genetic and metabolic engineering strategies have boosted the capacities of *Y. lipolytica*, making the yeast into a promising host for biotechnological production processes.

The objective of this study was to biosynthesize odd-chain FAs *de novo* from glucose without propionate supplementation. We constructed a modular metabolic pathway for synthesizing propionyl-CoA from oxaloacetate in *Y. lipolytica* and confirmed that accumulation of odd-chain FAs was increased. We also investigated whether it could be competitive to produce odd-chain FAs from propionyl-CoA via the methylcitrate pathway using the engineered strain. Additionally, we overexpressed the pyruvate dehydrogenase (PDH) complex in the cytosol to see if it could improve the conversion of α-ketobutyrate to propionyl-CoA. This work demonstrates that our metabolic engineering strategy for directing metabolic fluxes through specific pathways can enhance odd-chain FA production.

## Materials and Methods

### Strains and Media

Media and growth conditions for *E. coli* were previously described by Sambrook and Russell (2001); those for *Y. lipolytica* were previously described by Barth and Gaillardin (1997). Rich medium (YPD) and minimal glucose medium (YNB) were prepared as described elsewhere (Milckova et al., 2004). The YNB contained 0.17% (w/v) yeast nitrogen base (without amino acids and ammonium sulfate, YNBww), 0.5% (w/v) NH_4_Cl, 50 mM KH_2_PO_4_-Na_2_HPO_4_ (pH 6.8), and 2% (w/v) glucose. To complement strain auxotrophies, 0.1 g/L of uracil or leucine was added as necessary. To screen for hygromycin resistance, 250 μg/ml of hygromycin was added to the YPD. Solid media were prepared by adding 1.5% (w/v) agar.

### Construction of Plasmids and Strains (*E. coli* and *Y. lipolytica*)

We used standard molecular genetic techniques (Sambrook and Russell, 2001). Restriction enzymes were obtained from New England Biolabs (Ipswich, MA, USA). PCR amplification was performed in an Eppendorf 2720 Thermal Cycler with either Q5 High-Fidelity DNA Polymerase (New England Biolabs) or GoTaq DNA polymerases (Progema, WI, USA). PCR fragments were purified using a PCR Purification Kit (Macherey-Nagel, Duren, Germany), and plasmids were purified with a Plasmid Miniprep Kit (Macherey-Nagel).

The plasmids used in this study were constructed using Golden Gate assembly, as described in Celinska et al. (2017). The genes in the A, T, and H module were obtained via PCR using the genomic DNA of *Y. lipolytica* W29. Internal BsaI recognition sites were removed via PCR using the primers listed in Supplementary Table 1. The plasmids for each module included the Zeta sequence, the *URA3 ex* marker, and gene expression cassettes containing the *TEF1* promoter and *LIP2* terminator.

For the cytosolic PDH complex, all the genes were synthesized and cloned in the plasmid pUC57 by GenScript Biotech (New Jersey, US). Cytosolic *PDX1* was cloned into the expression plasmid (JME2563) using the BamHI and AvrII restriction sites. The other four genes were cloned into two plasmids (JME4774 and JME4775) using Golden Gate assembly.

To disrupt *PHD1*, the cassettes were constructed to include a promoter (*pPHD1*), a marker (*URA3* or *LEU2*), and a terminator (*TPHD1*), which allowed the ORF gene to be removed via homologous recombination, as described in Papanikolaou et al. (2013).

Gene expression and disruption cassettes were prepared by NotI digestion and transformed into *Y. lipolytica* strains using the lithium acetate method, as described previously (Barth and Gaillardin, 1997). Gene integration and disruption were verified via colony PCR using the primers listed in Supplementary Table 1. The replicative plasmid harboring the *Cre* gene (JME547; Table 1) was used for marker rescue (Fickers et al., 2003). After transformation with the *Cre* expression plasmid, the loss of the marker gene was verified on YNB with/without uracil. The loss of the replicative plasmid was checked using replica plating on YPD with/without hygromycin after culturing on YPD for 24 h. To construct the prototrophic strain, a *LEU2* fragment from plasmid JMP2563 was transformed. All the strains and plasmids used in this study are listed in Table 1.

**Table 1:** The plasmids and strains used in this study.

**Strain**	**Description**	**Abbreviation**	**References**
**Plasmid**			
GGE0004	TOPO-P2-*TEF1*		Celinska et al., 2017
GGE0009	TOPO-P3-*TEF1*		Celinska et al., 2017
GGE0014	TOPO-T1-*LIP2*		Celinska et al., 2017
GGE0015	TOPO-T2-*LIP2*		Celinska et al., 2017
GGE0020	TOPO-T1-3-*LIP2*		Celinska et al., 2017
GGE0021	TOPO-T2-3-*LIP2*		Celinska et al., 2017
GGE0028	pSB1C3		Celinska et al., 2017
GGE0029	pSB1A3		Celinska et al., 2017
GGE0038	TOPO-ZetaDOWN-NotI		Celinska et al., 2017
GGE0067	TOPO-ZetaUP-NotI		Celinska et al., 2017
GGE0081	TOPO-T3-*LIP2*		Celinska et al., 2017
GGE0082	TOPO-P1-*TEF1*		Celinska et al., 2017
GGE0085	TOPO-M-*URA3* ex		Celinska et al., 2017
GGE0376	TOPO-*AAT2*		This study
GGE0377	TOPO-*THR1*		This study
GGE0378	TOPO-*THR4*		This study
GGE0379	pJET-*ILV1*		This study
GGE0380	TOPO-*HOM3*		This study
GGE0381	TOPO-*HOM2*		This study
GGE0382	pJET-*HOM6*		This study
JME0547	pUC-*Cre*		Fickers et al., 2003
JME0740	pGEM-T-*PHD1* PUT		Papanikolaou et al., 2013
JME1811	pGEM-T-*PHD1* PLT		Papanikolaou et al., 2013
JME2563	JMP62-*LEU2*ex-pTEF1		Dulermo et al., 2017
JME4478	GGV-*URA3* ex-pTEF1-*AAT2*	Module A	This study
JME4479	GGV-*URA3* ex-pTEF1-*THR1*-pTEF-*THR4*-pTEF-*ILV1*	Module T	This study
JME4632	GGV-*URA3* ex-pTEF1-*HOM3*-pTEF1-*HOM2*-pTEF1-*HOM6*	Module H	This study
JME4774	GGV-*URA3* ex-pTEF1-yl*PDA1*-pTEF1-yl*PDB1*	Module P	This study
JME4775	GGV-*URA3* ex-pTEF1-yl*LPD1*-pTEF1-yl*LAT1*	Module P	This study
JME4776	JMP62-*LEU2* ex-pTEF1-yl*PDX1*	Module P	This study
***Y. lipolytica***			
JMY195	MATa *ura3-302 leu2-270 xpr2-322*	WT	Barth and Gaillardin, 1997
JMY2900	JMY195 *URA3 LEU2*	WT	Dulermo et al., 2014
JMY7201	JMY195 + GGV-*AAT2-URA3* ex	WT-A	This study
JMY7202	JMY195 + GGV-*AAT2*	WT-A	This study
JMY7639	JMY195 + GGV-*AAT2*-*URA3* ex + *LEU2*	WT-A	This study
JMY7203	JMY195 + GGV-*AAT2* + GGV-*THR1*-*THR4*-*ILV1*-*URA3* ex	WT-AT	This study
JMY7204	JMY195 + GGV-*AAT2* + GGV-*THR1*-*THR4*-*ILV1*	WT-AT	This study
JMY7353	JMY195 + GGV-*AAT2* + GGV-*THR1*-*THR4*-*ILV1* +GGV-*HOM3*-*HOM2*-*HOM6*-*URA3* ex	WT-ATH	This study
JMY7357	JMY195 + GGV-*AAT2* + GGV-*THR1*-*THR4*-*ILV1* +GGV-*HOM3*-*HOM2*-*HOM6*-*URA3* ex + *LEU2*	WT-ATH	This study
JMY7374	JMY195 + GGV-*AAT2* + GGV-*THR1*-*THR4*-*ILV1* +GGV-*HOM3*-*HOM2*-*HOM6*-*URA3* ex + *phd1*::*LEU2* ex	WT-ATH *phd1Δ*	This study
JMY7828	JMY195 + GGV-*AAT2* + GGV-*THR1*-*THR4*-*ILV1* +GGV-*HOM3*-*HOM2*-*HOM6*	WT-ATH	This study
JMY7640	JMY195 + GGV-*THR1*-*THR4*-*ILV1*-*URA3* ex	WT-T	This study
JMY7643	JMY195 + GGV-*THR1*-*THR4*-*ILV1*-*URA3* ex + *LEU2*	WT-T	This study
JMY7646	JMY195 + GGV-*HOM3*-*HOM2*-*HOM6*-*URA3* ex	WT-H	This study
JMY7649	JMY195 + GGV-*HOM3*-*HOM2*-*HOM6*-*URA3* ex + *LEU2*	WT-H	This study
JMY7824	Y195ATH+ylPDHcyto	WT-ATHP	This study
JMY3501	*Δpox1-6 Δtgl4* pTEF-*DGA2*-*LEU2*ex pTEF-*GPD1*-*URA3*ex	Obese	Lazar et al., 2014
JMY3820	*Δpox1-6 Δtgl4* pTEF-*DGA2* pTEF-*GPD1*	Obese	Lazar et al., 2014
JMY7206	JMY3820 + GGV-*AAT2*-*URA3* ex	Obese-A	This study
JMY7207	JMY3820 + GGV-*AAT2*	Obese-A	This study
JMY7208	JMY3820 + GGV-*AAT2* + GGV-*THR1*-*THR4*-*ILV1*-*URA3* ex	Obese-AT	This study
JMY7267	JMY3820 + GGV-*AAT2* + GGV-*THR1*-*THR4*-*ILV1*	Obese-AT	This study
JMY7412	MY3820 + GGV-*AAT2* + GGV-*THR1*-*THR4*-*ILV1* + GGV-*HOM3*-*HOM2*-*HOM6*-*URA3* ex + *LEU2*	Obese-ATH	This study
JMY7413	MY3820 + GGV-*AAT2* + GGV-*THR1*-*THR4*-*ILV1* + GGV-*HOM3*-*HOM2*-*HOM6*	Obese-ATH	This study
JMY7414	JMY7413 + *phd1::LEU2* ex	Obese-ATH *phd1Δ*	This study
JMY7417	JMY7413 + *phd1::LEU2* ex + *URA3*	Obese-ATH *phd1Δ*	This study
JMY7826	Y3820ATH+ylPDHcyto	Obese-ATHP	This study

### Culture Conditions for the Lipid Biosynthesis Experiments

The lipid biosynthesis experiments were carried out in minimal media, and the cultures were prepared as follows: an initial pre-culture was established by inoculating 10 mL of YPD medium in 50 mL Erlenmeyer flasks. Then, the pre-culture was incubated overnight at 28°C and 180 rpm. The resulting cell suspension was washed with sterile distilled water and used to inoculate 50 mL of minimal medium YNBD6 containing 0.17% (w/v) yeast nitrogen base (without amino acids and ammonium sulfate, YNBww, Difco), 0.15% (w/v) NH_4_Cl, 50 mM KH_2_PO_4_-Na_2_HPO_4_ buffer (pH 6.8), and 6% (w/v) glucose. This medium had been placed in 250 mL Erlenmeyer flasks. The cultures were then incubated at 28°C and 180 rpm.

### Lipid Determination

Lipids were extracted from 10 to 20 mg of freeze-dried cells and converted into FA methyl esters (FAMEs) using the procedure described by Browse et al. (1986). The FAMEs were then analyzed using gas chromatography (GC), which was carried out with a Varian 3900 instrument equipped with a flame ionization detector and a Varian FactorFour vf-23ms column, where the bleed specification at 260°C is 3 pA (30 m, 0.25 mm, 0.25 μm). The FAMEs were identified via comparisons with commercial standards (FAME32, Supelco) and quantified using the internal standard method, which involves the addition of 100 μg of commercial dodecanoic acid (Sigma-Aldrich). Commercial odd-chain FAs (Odd Carbon Straight Chains Kit containing 9 FAs, OC9, Supelco) were converted into their FAMEs using the same method employed with the yeast samples. They were then identified using GC and compared with the odd-chain FAs from the yeast samples.

To determine dry cell weight (DCW), 2 mL of the culture was taken from the flasks, washed, and lysophilized in a pre-weighed tube. The differences in mass corresponded to the mg of cells found in 2 mL of culture.

## Results

### Modular Pathway Engineering Was Used for Odd-Chain Fatty Acid Synthesis

**Figure 2:**
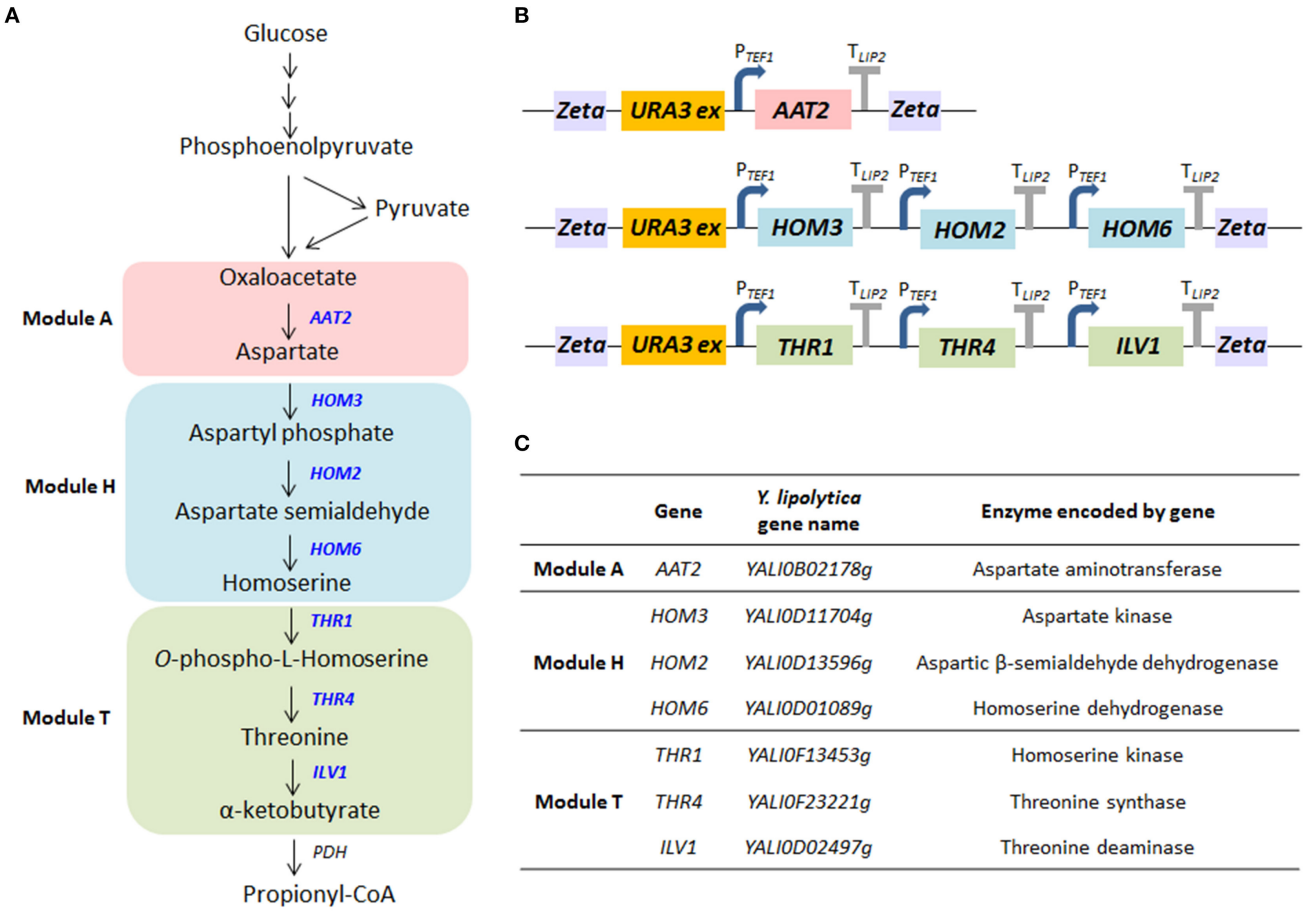
The biosynthetic pathway for propionyl-CoA. **(A)** The metabolic pathway by which propionyl-CoA is synthesized from glucose. The genes overexpressed in this study are shown in blue. Pyruvate produced by glycolysis is converted to oxaloacetate by pyruvate carboxylase, and oxaloacetate is converted to threonine through the biosynthesis of aspartate and homoserine. Threonine deaminase subsequently generates α-ketobutyrate from threonine. Then, α-ketobutyrate is converted to propionyl-CoA by the pyruvate dehydrogenase (PDH) complex. **(B)** Structure of the multigene modules encoding enzymes in the aspartate/α-ketobutyrate pathway that were constructed in this study. Each gene expression cassette included the native *TEF1* promoter and *LIP2* terminator. **(C)** The genes included in the modules and the enzymes they encode.

Propionyl-CoA is a key primer in the synthesis of odd-chain FAs. It can be synthesized using β-oxidation from direct precursors, propionate, or long-chain FAs. It can also be created from other metabolites via several metabolic pathways, such as the citramalate/2-ketobutyrate pathway, the aspartate/2-ketobutyrate pathway, the methylmalonyl-CoA pathway, the 3-hydroxypropionate pathway, and the isoleucine or valine degradation pathway (Supplementary Figure 1; Han et al., 2013; Lee et al., 2013). Here, we tested if the overexpression of the α-ketobutyrate pathway—which produces threonine as an intermediate—could increase levels of propionyl-CoA in *Y. lipolytica*. As shown in Figure 2A, the pathway eventually forms the amino acids aspartate, homoserine, and threonine from oxaloacetate. Then, threonine is deaminated to generate α-ketobutyrate, a reaction catalyzed by threonine dehydratase. Alpha-ketobutyrate is directly or sequentially converted into propionyl-CoA by the pyruvate dehydrogenase (PDH) complex or pyruvate oxidase, respectively. The upregulation of threonine has previously been used to boost propionyl-CoA availability in *E. coli*. Lee et al. showed that levels of odd-chain FAs could be increased by introducing the threonine biosynthesis pathway (which creates α-ketobutyrate from aspartate semialdehyde), especially when mutated homoserine dehydrogenase (thrA^*^, reduced feedback inhibition) was also expressed (Lee et al., 2013). The percentage of odd-chain FAs out of total FAs increased from <1 to 18% by overexpressing the threonine pathway in *E. coli*. The predominant odd-chain FA produced was pentadecanoic acid (C15:0).

In this study, we enhanced the extended threonine biosynthesis pathway (the aspartate/α-ketobutyrate pathway)—from oxaloacetate to α-ketobutyrate—by overexpressing seven genes (Figure 2A). There were three modules (Figures 2A,C): the aspartate synthesis module (module A), which included *AAT2*; the homoserine synthesis module (module H), which included *HOM3, HOM2*, and *HOM6*; and the threonine synthesis module (module T), which included *THR1, THR4*, and *ILV1*. While threonine and α-ketobutyrate (module T) are synthesized in the mitochondria in *S. cerevisiae*, the same may not be true in *Y. lipolytica*. While the locations of the relevant enzymes are as yet unknown in *Y. lipolytica*, predictive analyses (Supplementary Table 4) suggest enzyme location may differ between *S. cerevisiae* and *Y. lipolytica*. Because the module T enzymes were predicted to occur in the cytoplasm in *Y. lipolytica*, we used the original sequence of each gene in this study, as described in Supplementary Table 3. However, more research is needed to confirm enzyme locations in *Y. lipolytica*. The genes in each module were cloned into one plasmid using Golden Gate assembly (Figure 2B). They were expressed under the constitutive promoter p*TEF1*, and the expression cassette of each module was randomly integrated into the genome. Each module (A, T, and H) in the pathway was overexpressed in *Y. lipolytica* both individually and in tandem. The strain with the full modular pathway (ATH) was constructed by removing and reusing the *URA3* marker (Supplementary Figure 2). We verified gene integration using colony PCR with the primer set of promoters and the ORF gene (Supplementary Table 1).

### The Engineered Strain Could Produce Odd-Chain Fatty Acids Using Glucose as Its Sole Carbon Source

**Figure 3:**
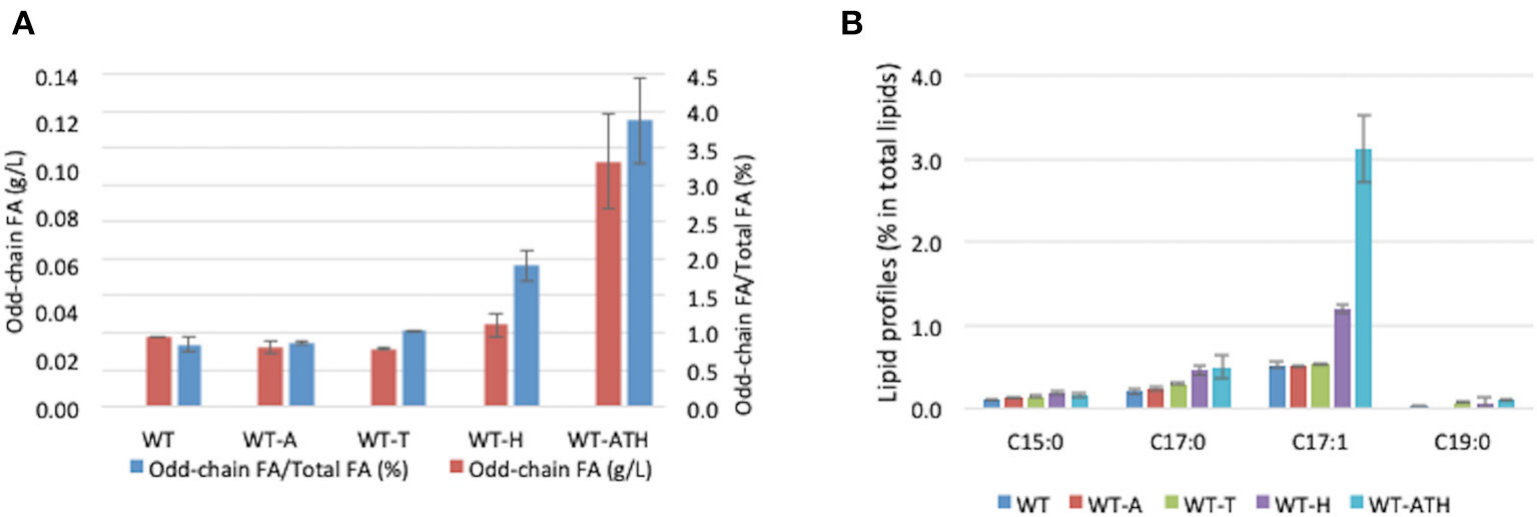
Odd-chain fatty acid production and profiles for the wild-type and engineered strains. **(A)** Odd-chain fatty acid (FA) titers (g/L) and the percentage of odd-chain FAs relative to total FAs in the wild-type (WT) and engineered strains. **(B)** Odd-chain FA profiles (percentage of each FA out of total FAs) of the WT strain and engineered strains. WT, control strain; WT-A, strain expressing module A; WT-T, strain expressing module T; WT-H, strain expressing module H; WT-ATH, strain expressing the full pathway (all three modules). The results represent the means and standard deviations for two independent experiments.

To determine whether the modular metabolic pathway was effective in producing odd-chain FAs, we evaluated the performance of the engineered strains overexpressing the individual modules and the entire pathway. The strains were cultivated in YNBD6 medium under nitrogen limitation conditions (C/N = 60), which have been found to positively influence lipid synthesis (Beopoulos et al., 2012; Ledesma-Amaro et al., 2016). For the WT-A strain, which overexpressed *AAT2*, and the WT-T strain, which overexpressed *THR*1, *THR4*, and *ILV1*, total lipid content (%, g/g DCW) was lower than in the wild-type (WT) strain; the percentage of odd-chain FAs out of total FAs was similar (Table 2). For the WT-H strain, which overexpressed *HOM3, HOM2*, and *HOM6*, this percentage was slightly greater (1.91%) than that in the WT strain (0.84%) (Table 2 and Figure 3A). For the WT-ATH strain, which overexpressed the entire pathway, odd-chain FA synthesis was significantly greater, odd-chain FA content (%, g/g DCW) was 3.8 times higher, and odd-chain FA titers (g/L) were 3.6 times higher than in the WT strain; the percentage of odd-chain FAs out of total FAs was 3.86%, which was 4.6 times higher than the value seen in the WT strain. These results indicate that the engineered aspartate/α-ketobutyrate pathway can supply propionyl-CoA; they also show that the full pathway is needed for effective odd-chain FA synthesis.

**Table 2:** Fatty acid (FA) production in the wild-type (WT) strain and the engineered strains after growth on YNBD6 medium for 120 h.

**Strain**	**DCW (g/L)**	**Lipid content %**		**Odd-chain FA /** **Total FA (%)**	**Lipid titer (g/L)**	
		**Total FA**	**Odd-chain FA**		**Total FA**	**Odd-chain FA**
WT	18.65 ± 0.15	19.13 ± 2.22	0.16 ± 0.00	0.84 ± 0.09	3.571 ± 0.442	0.029 ± 0.000
WT-A	17.30 ± 0.15	16.60 ± 1.05	0.14 ± 0.01	0.87 ± 0.03	2.873 ± 0.207	0.025 ± 0.003
WT-T	16.50 ± 0.00	14.41 ± 0.21	0.15 ± 0.00	1.03 ± 0.01	2.378 ± 0.035	0.024 ± 0.001
WT-H	16.30 ± 0.55	10.96 ± 0.09	0.21 ± 0.03	1.91 ± 0.21	1.788 ± 0.075	0.034 ± 0.005
WT-ATH	16.90 ± 0.25	15.73 ± 0.95	0.61 ± 0.13	3.86 ± 0.57	2.656 ± 0.121	0.103 ± 0.020

*The values represent the means and standard deviations for two independent experiments. DCW, dry cell weight*.

WT-ATH primarily produced C17:1 FAs (Figure 3B). This profile resembles that of an engineered *Y. lipolytica* strain that received propionate supplementation (Park et al., 2018). This finding implies that enhancing carbon flux through the α-ketobutyrate pathway can boost propionyl-CoA availability and odd-chain FA synthesis the same way that propionate supplementation can.

### Odd-Chain Fatty Acid Production Was Significantly Improved in a Lipid-Accumulating Strain

Once we had determined that the strain overexpressing the full modular pathway produced more odd-chain FAs, we wanted to boost their accumulation. Previously, we had engineered a *Y. lipolytica* strain, JMY3501, to accumulate large amounts of lipids (Lazar et al., 2014). We eliminated lipid degradation and remobilization in this obese strain by deleting the *POX* (*POX1-6*) genes (Beopoulos et al., 2008) as well as the *TGL4* gene, which encodes a triglyceride lipase (Dulermo et al., 2013). In addition, we overexpressed *DGA2*, which encodes the major acyl-CoA:diacylglycerol acyltransferase (Beopoulos et al., 2012), and *GPD1*, which encodes glycerol-3-phosphate dehydrogenase, in order to push and pull TAG biosynthesis (Dulermo and Nicaud, 2011; Tai and Stephanopoulos, 2013). Therefore, using JMY3501, we built a new obese strain that overexpressed our full modular pathway. It was called the obese-ATH strain. We then studied lipid production under the same conditions as before. As expected, total lipid accumulation was 2.29-fold greater in the obese-ATH strain than in the WT-ATH strain (Tables 2, 3). Interestingly, the obese-ATH strain also accumulated more odd-chain FAs: the percentage of odd-chain FAs out of total FAs was 5.64% in the obese-ATH strain vs. 3.86% in the WT-ATH strain. The obese-ATH strain produced 0.36 g/L of odd-chain FAs, which is 7.2 times greater than the amount produced by the regular obese strain. The obese-ATH strain and the WT-ATH strain differed in their even-chain FA profiles (Table 4). The obese-ATH had slightly higher levels of C16:0 and slightly lower levels of C18:1, a common pattern seen in strains with the obese background regardless of the carbon source (Lazar et al., 2014; Ledesma-Amaro et al., 2016).

**Table 3:** Fatty acid (FA) production in the obese strain and the obese-ATH strain after growth on YNBD6 medium for 120 h.

**Strain**	**DCW (g/L)**	**Lipid content %**		**Odd-chain FA /** **Total FA (%)**	**Lipid titer (g/L)**	
		**Total FA**	**Odd-chain FA**		**Total FA**	**Odd-chain FA**
Obese	19.20 ± 0.04	37.11 ± 0.14	0.25 ± 0.00	0.68 ± 0.01	7.125 ± 0.012	0.049 ± 0.001
Obese-ATH	17.62 ± 0.03	36.02 ± 0.39	2.03 ± 0.05	5.64 ± 0.06	6.347 ± 0.058	0.358 ± 0.009

*The values represent the means and standard deviations for two independent experiments. DCW, dry cell weight*.

**Table 4:** Comparison of the lipid profiles (% of each FA) of the WT-ATH strain and the obese-ATH strain.

**Strain**	**C15:0**	**C16:0**	**C16:1**	**C17:0**	**C17:1**	**C18:0**	**C18:1**	**C18:2**	**C19:0**
WT-ATH	0.16 ± 0.03	8.31 ± 0.75	6.13 ± 0.00	0.50 ± 0.14	3.11 ± 0.40	5.96 ± 0.68	61.31 ± 2.43	11.02 ± 0.26	0.10 ± 0.00
Obese-ATH	0.40 ± 0.00	13.07 ± 0.01	7.25 ± 0.11	0.76 ± 0.00	4.26 ± 0.06	5.00 ± 0.17	50.12 ± 0.27	14.11 ± 0.16	0.22 ± 0.13

*The values represent the means and standard deviations for two independent experiments*.

### The Disruption of *PHD1* in the Methylcitrate Cycle Did Not Increase Odd-Chain Fatty Acid Production

**Figure 4:**
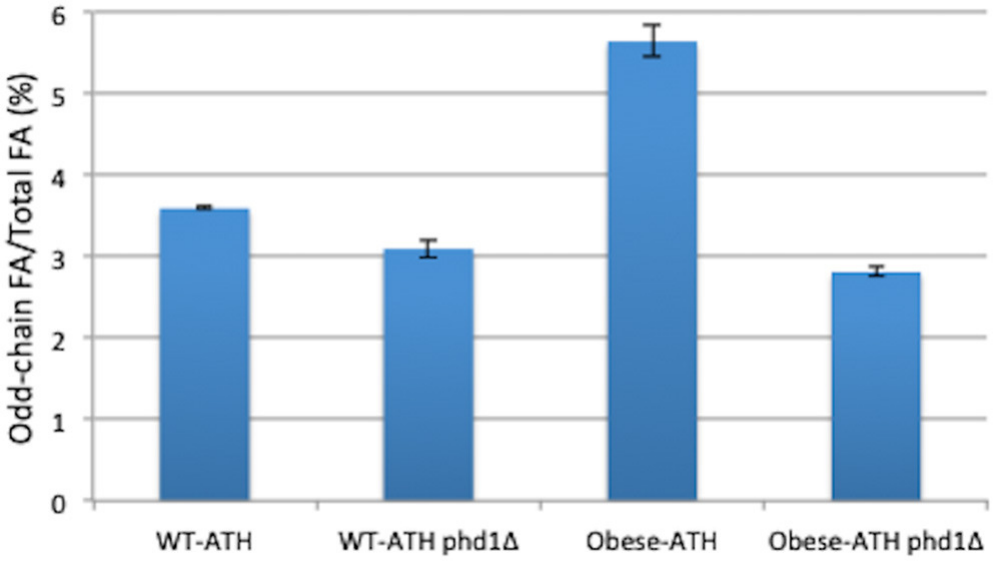
Odd-chain fatty acid production in the *PHD1*-disrupted strains. The percentage of odd-chain fatty acids (FAs) relative to total FAs in the *PHD1*-disrupted strains and their respective controls. The results represent the means and standard deviations for two independent experiments.

In a previous study, we inactivated *PHD1*, the gene that encodes 2-methylcitrate dehydratase, which catalyzes the conversion of 2-methyl citrate to 2-methyl-*cis*-aconitate in the methylcitrate cycle; we showed that the resulting higher levels of propionyl-CoA could be used to synthesize greater amounts of odd-chain FAs (Park et al., 2018). To investigate whether the inhibition of the methylcitrate cycle—via the deletion of *PHD1*—could further improve the accumulation of odd-chain FAs, we disrupted the *PHD1* gene in both the WT-ATH strain and the obese-ATH strain. The two *phd1*Δ strains displayed higher total lipid content compared to their relative controls (Supplementary Table 2), a result that was also demonstrated in Papanikolaou et al. (2013); however, they also displayed lower percentages of odd-chain FAs out of total FAs (Figure 4). This latter negative effect was significantly more pronounced in the obese-ATH *phd1*Δ strain than in the WT-ATH *phd1*Δ strain. For the obese-ATH *phd1*Δ strain, this figure dropped by 50%, and levels of odd-chain FAs were 67% of those seen in the relative control (0.24 vs. 0.36 g/L). These results suggest that disrupting the methylcitrate cycle does not provide the benefits seen previously (Park et al., 2018) when strains are already overexpressing the aspartate/α-ketobutyrate pathway.

The action of 2-methylcitrate dehydratase is restricted to the mitochondria. Consequently, disabling this enzyme does not directly improve cytosolic levels of propionyl-CoA. In the previous studies showing that the disruption of *PHD1* boosted odd-chain FA synthesis (Papanikolaou et al., 2013; Park et al., 2018), there was also propionate supplementation. Taken together, these findings suggest that, when the methylcitrate cycle is disrupted, the overexpression of the aspartate/α-ketobutyrate pathway has a weaker effect on propionyl-CoA levels than does propionate supplementation.

### Overexpression of the Cytosolic Pyruvate Dehydrogenase (PDH) Complex Improved Lipid Synthesis but Reduced Odd-Chain Fatty Acid Production

The PDH complex consists of three main catalytic components: E1 (pyruvate dehydrogenase, encoded by *PDA1* and *PDB1*), E2 (dihydrolipoamide acetyltransferase, encoded by *LAT1*), and E3 (dihydrolipoamide dehydrogenase, encoded by *LPD1*). There is a fourth component (protein X, encoded by *PDX1*) that binds to and positions E3 relative to E2. In *Y. lipolytica*, the PDH complex is found in mitochondria and catalyzes the pathway from pyruvate to acetyl-CoA. A few studies have examined the functional expression of the PDH complex in *Y. lipolytica*. One study attempted to overexpress the direct pathway from pyruvate to acetyl-CoA in a coordinated manner (Markham et al., 2018), and another study showed that the individual overexpression of *PDA1* (shared subunits with α-ketoglutarate dehydrogenase) improved α-ketoglutarate production (Guo et al., 2014).

Here, we wanted to redirect the aspartate/α-ketobutyrate pathway to produce propionyl-CoA via the PDH complex, which has already been shown to be possible in *E. coli* (Danchin et al., 1984; Tseng and Prather, 2012; Lee et al., 2013). The PDH complex was built so it could be associated with the full modular pathway (ATH) and could provide a pool of propionyl-CoA in the cytosol where lipid synthesis takes place. The mitochondrial targeting sequences (MTSs) of each gene were predicted using MitoProt (Claros and Vincens, 1996), and the gene sequences used in this study are described in Supplementary Table 3. We then created the obese-ATHP strain by overexpressing the cytosolic PDH subunits in the obese-ATH strain. Next, to explore lipid accumulation dynamics, the strain was grown in YNBD6 with lipoic acid, which is required for cytosolic PDH activity in yeast (Kozak et al., 2014). Compared to the control strain, the obese-ATHP strain had significantly lower levels of odd-chain FAs (a 3.8 times lower percentage of odd-chain FAs out of total FAs); however, total lipid levels were higher (Table 5). The increase in lipid production might have resulted from the increased levels of cytosolic acetyl-CoA resulting from the overexpression of the PDH complex. A similar strategy was utilized in *Saccharomyces cerevisiae*, where PDH was introduced into the cytosol and was found to increase levels of acetyl-CoA (Kozak et al., 2014; Lian et al., 2014) and of the target compound of interest. Since acetyl-CoA is a key precursor in the production of both even- and odd-chain FAs, increasing levels of acetyl-CoA promotes lipid synthesis in general. However, the substantially lower levels of odd-chain FAs in the obese-ATHP strain implies that the PDH complex shows greater specificity for pyruvate than for α-ketobutyrate. The higher Km value of α-ketobutyrate compared to that of pyruvate has been seen elsewhere, such as in *E. coli* (Bisswanger, 1981), *Neurospora crassa* (Harding et al., 1970), and mammalian cells (Bremer, 1969). Therefore, it is important to explore enzyme engineering strategies that modify substrate specificity or that introduce other enzymes that can convert acetyl-CoA to propionyl-CoA with a view to further improving odd-chain FA production via a threonine-based upregulation strategy.

**Table 5:** Fatty acid (FA) production in the obese-ATH strain and the obese-ATHP strain after growth on YNBD6 medium for 120 h.

**Strain**	**DCW (g/L)**	**Lipid content %**		**Odd-chain FA /** **Total FA (%)**	**Lipid titer (g/L)**	
		**Total FA**	**Odd-chain FA**		**Total FA**	**Odd-chain FA**
Obese-ATH	15.68 ± 0.02	29.23 ± 0.01	1.59 ± 0.03	5.44 ± 0.10	4.582 ± 0.010	0.249 ± 0.005
Obese-ATHP	15.25 ± 0.25	33.89 ± 1.06	0.48 ± 0.03	1.42 ± 0.03	5.171 ± 0.246	0.073 ± 0.005

*The values represent the means and standard deviations for two independent experiments. DCW, dry cell weight*.

## Discussion

In this study, we developed a synthetic biological strategy for the *de novo* production of odd-chain FAs in *Y. lipolytica*. It is important to note that the wild-type *Y. lipolytica* strain produces only negligible amounts of odd-chain FAs even though it has an excellent capacity to accumulate large quantities of lipids. Several studies have shown that propionate supplementation can increase the production of odd-chain FAs (Fontanille et al., 2012; Kolouchová et al., 2015; Park et al., 2018). However, research has yet to explore the *de novo* production of odd-chain FAs from sugars in *Y. lipolytica*.

The overexpression of the aspartate/α-ketobutyrate pathway (from oxaloacetate to homoserine and threonine) via Golden Gate assembly resulted in higher levels of odd-chain FAs being produced from glucose. The best strain generated a level of odd-chain FAs, 0.36 g/L in flask that is the highest to date to be achieved in *Y. lipolytica* without propionate supplementation. Furthermore, it is comparable to the levels seen in a previous study of ours that employed propionate supplementation, where odd-chain FAs titers were 0.14 and 0.57 g/L in the wild-type strain and the obese strain, respectively (Park et al., 2018). To further increase the amount of propionyl-CoA produced via the overexpression of threonine synthesis, we constructed a cytosolic pyruvate dehydrogenase (PDH) complex. Because of the lower specificity of the PDH complex for α-ketobutyrate vs. pyruvate, the engineered strain generated lower levels of propionyl-CoA than did the relative control; however, the increased levels of acetyl-CoA in the engineered strain led to larger amounts of total FAs. This study is the first to describe the functional expression of the native PDH complex in the cytosol in *Y. lipolytica*, an approach that could also be employed to produce acetyl-CoA-derived compounds, such as polyhydroxybutyrates, isoprenoids, sterols, polyketides, polyphenols, alkanes, and alkenes (Nielsen, 2014).

Before the approach described here can be applied at industrial scales, certain issues must be resolved. It is necessary to perform research in which combinatorial pathway analysis (Lütke-Eversloh and Stephanopoulos, 2008), targeted-proteomics analysis (Redding-Johanson et al., 2011), and genome-scale metabolic network modeling (Xu et al., 2011) are used to identify bottlenecks and enhance metabolic fluxes to propionyl-CoA and odd-chain FAs. The results of the PDH overexpression experiment suggest that acetyl-CoA is a competitive precursor to propionyl-CoA in odd-chain FA synthesis and is also a precursor in lipid synthesis. Therefore, better balancing the pools of acetyl-CoA and propionyl-CoA could be key in further increasing odd-chain FA content. One way to improve odd-chain FA synthesis is to introduce enzymes—such as CoA transferase—that redirect CoA moieties from acetyl-CoA to propionyl-CoA (Yang et al., 2012) or that have greater specificity for 3-oxovaleryl-ACP than for acetoacetyl-CoA (Slater et al., 1998). Overall, this work has shown that applying synthetic biological engineering strategies in *Y. lipolytica* to improve odd-chain FA production could be useful in a wide range of pharmaceutical and industrial contexts.

## Data Availability Statement

The raw data supporting the conclusions of this article will be made available by the authors, without undue reservation, to any qualified researcher.

## Author Contributions

YP, RL-A, and J-MN planned the study. YP designed and carried out the experiments and drafted the manuscript. All the authors revised and approved the final manuscript.

### Conflict of Interest

The authors declare that the research was conducted in the absence of any commercial or financial relationships that could be construed as a potential conflict of interest.
