# Supplementary material for: De novo Biosynthesis of Odd-Chain Fatty Acids in Yarrowia lipolytica Enabled by Modular Pathway Engineering
Source: Front Bioeng Biotechnol. 2020 Jan 22;7:484. doi: 10.3389/fbioe.2019.00484 (PMC6987463; doi:10.3389/fbioe.2019.00484)
Supplement: Supplementary file 1 [file Table_1.docx]

**Supplementary Material**

**Title**

*De novo* biosynthesis of odd-chain fatty acids in *Yarrowia lipolytica* enabled by modular pathway engineering

**Authors and Affiliations**

Young-kyoung Park^1^, Rodrigo Ledesma-Amaro^2^, Jean-Marc Nicaud^1^

1. UMR1319, Institut Micalis, INRA, AgroParisTech, Université Paris-Saclay, France

2. Department of Bioengineering, Imperial College London, London, UK

**Corresponding authors**

Young-kyoung Park

Rodrigo Ledesma-Amaro

**Email address**

Young-Kyoung Park: YoungKyoung.Park@inra.fr

Rodrigo Ledesma-Amaro: r.ledesma-amaro@imperial.ac.uk

Jean-Marc Nicaud:jean-marc.nicaud@inra.fr

**Supplementary Figure 1. Metabolic pathways leading to propionyl-CoA synthesis.**

(The dashed arrows indicate multiple reactions.)

**
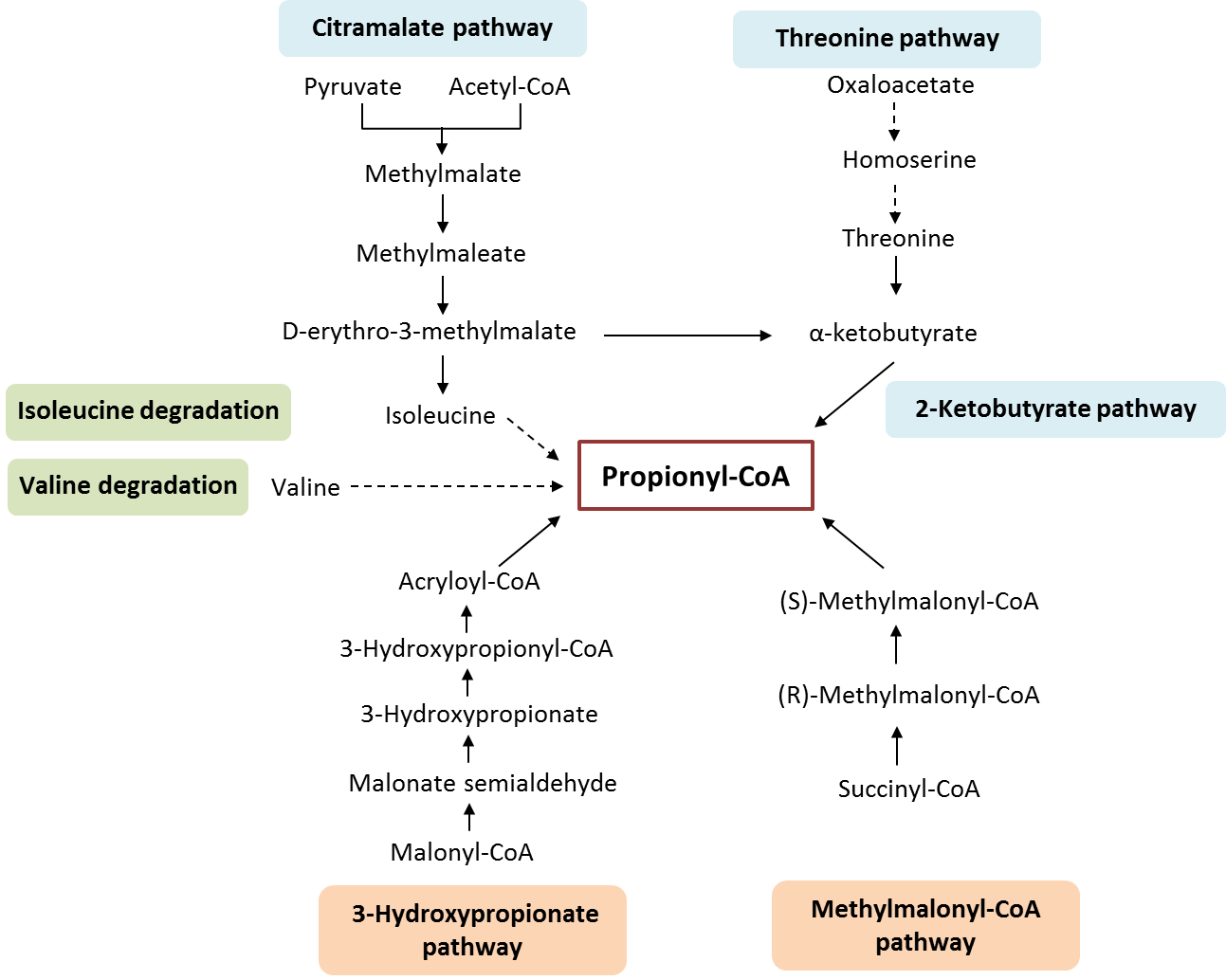
**

**Supplementary Figure 2. Construction of the *Y. lipolytica* strain containing the full modular pathway (ATH).**

The wild-type strain (JMY195) was used as the parental strain. Each plasmid was digested by NotI before transformation for genome integration. After the integration of module A (JME4478), the *URA3* marker gene was excised by transformation with the *Cre* plasmid (JME547), as described in Fickers et al. (2003). Modules T and H were then sequentially integrated in the same way. To construct the prototrophic strain, the *LEU2* gene fragment from JME2563 was transformed into a truncated *LEU2* locus in the parental strain for complementation.

**
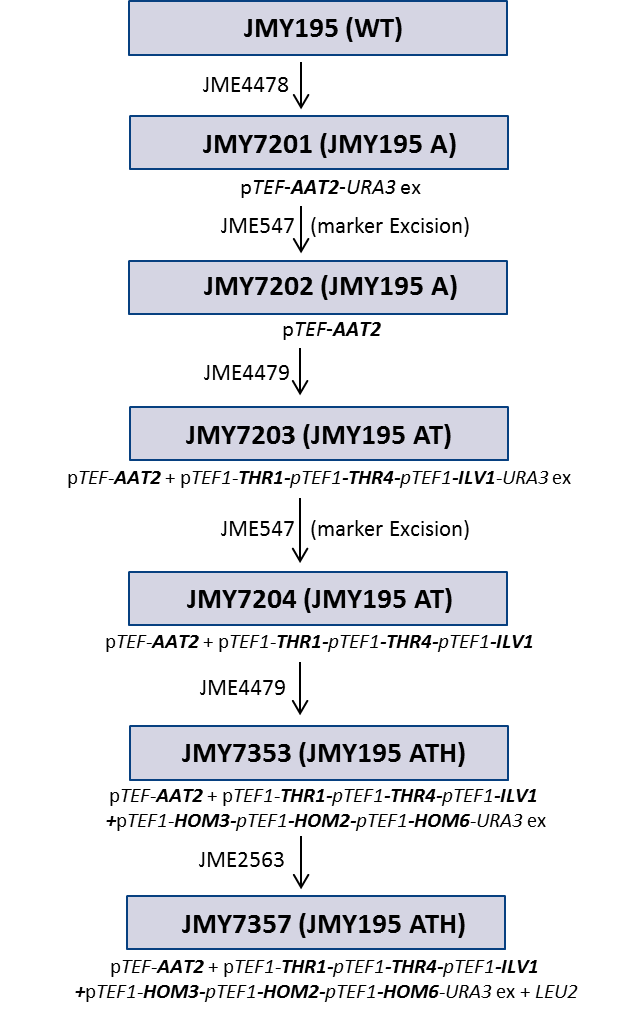
**

**Supplementary Table 1.** The sequence of primers used in this study.

| Name | Sequence (5’ - 3') | Use |
| --- | --- | --- |
| GGP_AAT2_A_F | GGTCTCTAATGCTCCGAACCATCGCC | Golden Gate assembly |
| GGP_AAT2_A_R | GGTCTCTTAGACTATTTGGTCACCTCGTAGATAGCCTG | Golden Gate assembly |
| GGP_THR1_A_F | GGTCTCTAATGTCTCGAAAGTTTGAAATTTCTGTGCCCGCGTCCTCGGC | Golden Gate assembly |
| GGP_THR1_A_R | GGTCTCTTAGATTAGAGCTGCTTCACGTTGGAACC | Golden Gate assembly |
| THR1_BsaI_del_1_F | ATGTCTCGAAAGTTTGAAATTTCTGTGC | Remove internal BsaI site |
| THR1_BsaI_del_1_R | GGCCGAGGCGGTGGACACTTCAAAG | Remove internal BsaI site |
| THR1_BsaI_del_2_F | CTTTGAAGTGTCCACCGCCTCGGCC | Remove internal BsaI site |
| THR1_BsaI_del_2_R | TTAGAGCTGCTTCACGTTGGAACCG | Remove internal BsaI site |
| GGP_THR4_B_F | GGTCTCTACAATGACCACCTACTTTTCCACGCG | Golden Gate assembly |
| GGP_THR4_B_R | GGTCTCtATCCTTACTGCTTCTTCTCCTTGGCCAG | Golden Gate assembly |
| THR4_BsaI_del_1_F | ATGACCACCTACTTTTCCACGC | Remove internal BsaI site |
| THR4_BsaI_del_1_R | CATCCTTAATAGTTTCGAGAGTCTGC | Remove internal BsaI site |
| THR4_BsaI_del_2_F | GCAGACTCTCGAAACTATTAAGGATG | Remove internal BsaI site |
| THR4_BsaI_del_2_R | GTAGCCCTCCAGGCCCTTGAGG | Remove internal BsaI site |
| THR4_BsaI_del_3_F | CCTCAAGGGCCTGGAGGGCTAC | Remove internal BsaI site |
| THR4_BsaI_del_3_R | TTACTGCTTCTTCTCCTTGGCCAGC | Remove internal BsaI site |
| GGP_ILV1_C_F | GGTCTCTCCACAATGTCCGAACCCGACTATCTGAAG | Golden Gate assembly |
| GGP_ILV1_C_R | GGTCTCTGTACTTACTTCATAAACTGCTTGTACACCACATTG | Golden Gate assembly |
| ILV1_BsaI_del_1_F | ATGTCCGAACCCGACTATCTGAAG | Remove internal BsaI site |
| ILV1_BsaI_del_1_R | GCATCATAAGTTTCCACGGCAATG | Remove internal BsaI site |
| ILV1_BsaI_del_2_F | GAAACTTATGATGCATGTGCTCTGAAAC | Remove internal BsaI site |
| ILV1_BsaI_del_2_R | CAGGCCAGCCACCGACAGAGCAC | Remove internal BsaI site |
| ILV1_BsaI_del_3_F | CGGTGGCTGGCCTGAAGAAGTAC | Remove internal BsaI site |
| ILV1_BsaI_del_3_R | TTACTTCATAAACTGCTTGTACACCACATTGTCTGTC | Remove internal BsaI site |
| GGP_HOM3_A_F | GGTCTCTAATGACCTGGATCGTCCAAAAGTTC | Golden Gate assembly |
| GGP_HOM3_A_R | GGTCTCTTAGACTAGATTTTCCGTCTAGGACACAACAAGC | Golden Gate assembly |
| GGP_HOM2_B_F | GGTCTCTACAATGGTCAAGACTAAAAAGGCTGGAGTTC | Golden Gate assembly |
| GGP_HOM2_B_R | GGTCTCTATCCCTAGATGAGATTCTTCTTCAGCAGAGTCTCG | Golden Gate assembly |
| HOM2_BsaI_del_1_F | ATGGTCAAGACTAAAAAGGCTGGAG | Remove internal BsaI site |
| HOM2_BsaI_del_1_R | CACCACCAGGCCGGCG | Remove internal BsaI site |
| HOM2_BsaI_del_2_F | GACGCCGGCCTGGTGGTGATTTC | Remove internal BsaI site |
| HOM2_BsaI_del_2_R | GGCAGACACCTTGAGCTCCGAGTCGGGAATGTTCTCAAAGGTGGCTCCGTCGGCGCTGACGTGGCCCAGAATCTTCTTAGTTTCCCAC | Remove internal BsaI site |
| HOM2_BsaI_del_3_F | GTGGGAAACTAAGAAGATTCTGGGCCACGTCAGCGCCGACGGAGCCACCTTTGAGAACATTCCCGACTCGGAGCTCAAGGTGTCTGCC | Remove internal BsaI site |
| HOM2_BsaI_del_3_R | CTAGATGAGATTCTTCTTCAGCAGAGTCTCGGCAATCAG | Remove internal BsaI site |
| GGP_HOM6_C_F | GGTCTCTCCACAATGTCCAAGGCTGTTAACATTGCTATTATC | Golden Gate assembly |
| GGP_HOM6_C_R | GGTCTCTGTACCTATTTGGCAAGTCGCTGGGC | Golden Gate assembly |
| HOM6_BsaI_del_1_F | ATGTCCAAGGCTGTTAACATTGCTATTATCGG | Remove internal BsaI site |
| HOM6_BsaI_del_1_R | CCCTCGACAGTTTCGACCTCGTCACCG | Remove internal BsaI site |
| HOM6_BsaI_del_2_F | CGGTGACGAGGTCGAAACTGTCGAGGG | Remove internal BsaI site |
| HOM6_BsaI_del_2_R | CTATTTGGCAAGTCGCTGGGCAGC | Remove internal BsaI site |
| PDA1-Rev | CGTTAGTTCTTAAAGTAGTAGTCCTCGG | Verification of gene |
| PDB1-Rev | CTACTCCTCAATGTAGAGGGCGTC | Verification of gene |
| LAT1-Rev | CTACAACAACATCTCAATGGGGTTTTC | Verification of gene |
| LPD1-rev | CTTAAAAGTGGATGGCCTTGTCGTAAG | Verification of gene |
| PDX1-Rev | CTACTCCTCCACAGTCAAATGATAC | Verification of gene |
| ZetaUp_Intern_Fw | TATCTTCTGACGCATTGACCAC | Verification of assembly |
| URA3M_Intern_Fw | CATCCAGAGAAGCACACAGG | Verification of assembly |
| URA3M_Intern_Rv | CAACTAACTCGTAACTATTACC | Verification of assembly |
| P-TEF_Intern_Fw | TCTGGAATCTACGCTTGTTCA | Verification of assembly |
| P_TEF_Intern_Rv | CTTAACGATTTCGGGTGTGAGT | Verification of assembly |
| T_Lip2_Intern_Fw | TGCGTTCCTCTAAGACAAATC | Verification of assembly |
| T_Lip2_Intern_Rv | GATTTGTCTTAGAGGAACGCATA | Verification of assembly |
| ZetaDown_Intern_Rv | GGTAACGCCGATTCTCTCTG | Verification of assembly |

**Supplementary Table 2.** Comparison of fatty acid (FA) production in the *PHD1*-disrupted strains and their relative controls after growth on YNBD6 for 120 h. The values represent the means and standard deviations for two independent experiments.

| Strain | DCW (g/L) | Lipid content % | | Odd FA  /Total FA (%) | Lipid titer (g/L) | |
| --- | --- | --- | --- | --- | --- | --- |
|  |  | Total FA | Odd FA |  | Total FA | Odd FA |
| WT-ATH | 14.03 ± 0.47 | 17.96 ± 0.16 | 0.65 ± 0.01 | 3.59 ± 0.02 | 2.519 ± 0.106 | 0.091 ± 0.004 |
| WT- ATH *phd1*Δ | 13.46 ± 0.07 | 18.46 ± 0.39 | 0.57 ± 0.01 | 3.10 ± 0.11 | 2.484 ± 0.065 | 0.077 ± 0.001 |
| Obese-ATH | 17.62 ± 0.03 | 36.02 ± 0.39 | 2.03 ± 0.05 | 5.64 ± 0.20 | 6.347 ± 0.058 | 0.358 ± 0.009 |
| Obese-ATH *phd1*Δ | 19.33 ± 0.02 | 43.37 ± 0.05 | 1.22 ± 0.02 | 2.81 ± 0.05 | 8.384 ± 0.018 | 0.236 ± 0.004 |

**Supplementary Table 3.** The sequences of the genes used in this study.

| **Gene** | **Sequence** |
| --- | --- |
| *AAT2 (YALI0B02178g)* | *GGTCTCtAATGCTCCGAACCATCGCCCGAACCCACGCTGTGGCCGCCACCAAGGTCGTGCGAGCCTCCACTTTTTCTGCCACCCCCGCCGCCTCTTTTGTGCGATTCCAGTCCGTCTGGGCCAAGGTCCCCCAGGGTCCCCCCGACGCCATTCTCGGAATCACCGAGGCGTTCAAGAAGGACGCCTTTGAGCAGAAGATCAACCTCGGTGTTGGCGCCTACCGAGATGACGGCGGAAAGCCCTTCGTTCTTCCCTCCGTCCGAGAGGCCGAGAAGGAGGTGGTGAACAAGGCCCTCGACAAGGAGTACGCCCCCATCACCGGAGTCCCCGCCTTCACCAAGGCTGCTGCCGAGCTCGCTTACGGCGCCGACTCCCCCGCCGTCCTCGAGGACCGAATTGCCATCACCCAGACCATCTCCGGTACCGGTGCTCTGCGAATCGGAGCCGAGTTCCTCAACAAGTTCTACTCCTCCAAGAAGATTCTGCTCCCCCAGCCTTCTTGGGCTAACCACAAGGCCGTTTTCACCGCCGCCGGCCTCGAGCCCGCCACCTACCGGTACTACGACCCCAAGAACATTGCCCTCGACTTTGAGGGTCTGCTCGCCGACCTGGAGGCTGCCCCCAACGGAACCGCCGTCCTTCTGCACGCCTGTGCCCACAACCCCACCGGTGTTGACCCCACTCCCGAGCAGTGGCGAAAGATTGAGGAGGTCGTCAAGGCCAAGGGCCACTTCCCCTTCTTCGACATGGCCTACCAGGGCTTTGCCACCGGTGACGTCAACCGAGACGCCTACCCCATCCGATACTTTGTCGAGCAGGGCCACGAGGTTGCTCTGTGCCAGTCCTTCGCCAAGAACATGGGTCTTTACGGTGAGCGAGTCGGTGCTTTCTCTCTTGTCTGCCAGGACACCGCCGAGAAGAACCGAGTTGACTCTCAGCTGAAGATTATCATCCGACCCTTCTACTCCAACCCCCCCGTCCACGGTGCCCGAATCGCCGCCACCATCCTCAACAACCCCGAGCTCAAGAAGCAGTGGCTCGGTGAGGTCAAGCAGATGGCCGACCGAATGATCAAGATGCGAGCTCTGCTCAAGGAGAACCTCGAGAGACTTGGCTCCCAGCAGAACTGGGACCACGTCACCTCTCAGATTGGTATGTTCTGCTACACCGGTCTGTCTCCTGAGCAGGTTGAGCGACTTGCCAAGGAGTTCTCCGTCTACGGAACCAAGGACGGCCGAATCTCCATTGCCGGAATCACCTCTCAGAACGTCGGCCGACTGGCCCAGGCTATCTACGAGGTGACCAAATAG* |
| *HOM3 (YALI0D11704g)* | *ATGACCTGGATCGTCCAAAAGTTCGGAGGAACATCGGTCGGCAAGTTCCCCAAAAACATCTGCAACGACATTGTGCAGCAGTACTCGCAGTCTTACGACGTGGCTGTGGTCTGCTCAGCCCGATCTACCGGAACCAAGGCCGAGGGAACCACCACCCGTCTTATTGCTGCTGCTGACTCTGCTCTTCTCGGATCAGACGCCTACAACCCCATCATTGAGTCTATCCGAGAAGACCATCTTGCAGCTGCTAAGCGTGACGTCAAGAACCCCGAACTGCTCGAAAACCTCAATGCCGACATCAACGGCGAGTGCGACCAGCTGTTGCGAATCCTGGCTGCTGCTGAAATCATTTCTGAGATCTCCCCCCGAACACTCGACTCCATCATGGCCATTGGGGAGAAGCTCAGTTGCATGTACATGACTGCAGTTATGCGGGACGCTGGTGTTAACGCACGGTACTTTGACCTGTCTCACGCAGTTACCACCACCAATGCCGAAGATCCCAAGTTCTATTCGGACCTCGGACGTGTTCTGGGCGAGATCATCACTCTTCCCGGCTCTCCTAACACGGACTCTGCTCCTGTCAAGGGAAACATGGTGCCCGTGTTGACCGGTTTCTTTGGCCCCGTCAAGGGAGGCCTCCTAAGCCAGATCGGACGAGGATACACCGATCTGTGTGCTGCTCTGGTGGCTGTGGGTCTGGACGCCAAGGAGCTTCAGATCTGGAAAGAAGTCGATGGTGTCTTCACTGCCGATCCCAGAAAGGTGTCCACTGCCCGTCTGCTGCCCATCATTACCCCCGAGGAGGCCGCTGAGTTGACCTACTACGGATCTGAAGTCATCCACCCCTTCACCATGGAGCAGGTCATTAAGGCCCATATTCCTATCCGAATCAAAAACGTGGAAAACCCCCTGGGAGGAGGAACTATCATCTACCCATCTGCTGACGGCTCGGGCAACCTGAAAACGAAAAACAATGTCCAGGATCTGGCTTCTTCCACCGACTCGCTAACTTCTCTGTCCTCTGGCTCATTCACTCCCCAGGTGTCTGCTCCTGTAGATGACGAGAAGAAGCCTACAGCTGTCACTACCAAATCGAATATCACCGTTCTCAACGTTCATTCGAATAAGCGAACCAAGAGTCATGGCTTCTTGAACAAGATCTTTGCTACTCTCGACCAGCAGAAACTTGTCGTCGATCTCATCTCCACTTCTGAGGTCCATGTGTCGATGGCCTTCCATGCTCCCGACTCCAACCTCAAGCAGGCTGTGGAGGAGCTGCGAAAGTACGGCACAGTAGATGTGAAGCGAGGCATGACCATTGTGTCGCTGATCGGAACGAGAATGAAGGCCATGGTCGGATGTGCAGGCATGTTCTTCTCTACTCTGGCCCAGGCAGGTATTAACATTGAGATGATTTCTCAGGGAGCCAACGAAATTAACATCTCGTGTGTTATTGAGGAAAAAGACGCGCTGAAGGCGTTGAACGTCATCCACCAGGGCTTGTTGTGTCCTAGACGGAAAATCTAG* |
| *HOM2 (YALI0D13596g)* | *ATGGTCAAGACTAAAAAGGCTGGAGTTCTCGGTGCCACCGGATCTGTGGGCCAGCGATTCATTCTGCTGCTCTCGGAGCACCCCGAGTTCGAGCTGTCTGTTCTGGGAGCTTCTTCTCGATCTGCCGGCAAGAAGTACGTTGATGCTTGTGACTGGAAGCAGACGGATGTTCTCCCCGAGGCTGCTGGCCAGACCGTGGTCAAGGAGTGCACTCCCGAGAACTTTGCCGAGTGCGACGTGGTCTTTTCCGGCCTGGACGCCGACTACGCTGGTGACATTGAGAAGGCTTTTGTTGACGCCGGCCTGGTGGTGATTTCCAACGCCAAGAACTACCGACGAGAGCCTACCGTGCCCCTGGTTGTCCCCACCGCCAACTCCGAGCACCTGGACTGCTTTTCCGAGCGAGTGGTTGCTGCCCGAAAGGCTGGCAAGAAGCAGGGCTACCAGATCTGCAACTCCAACTGCTCTACCGCCGGCCTTGTGATCCCTCTCAAGGCTCTGGTCGACGCCTTTGGCCCCATTGACAAGGTCATTGTCACAACCATGCAGGCCGTTTCCGGTGCTGGCTTCTCTCCCGGTGTCCCCTCCATGGATGTTCTCGATAACCTCATCCCTTACATTTCCGGCGAGGAGGACAAGATGGAGTGGGAAACTAAGAAGATTCTGGGCCACGTCAGCGCCGACGGAGCCACCTTTGAGAACATTCCCGACTCGGAGCTCAAGGTGTCTGCCACCTGCAACCGAGTGGCTGTCATTGACGGCCACACCGAATGTGTCGCCTTCTCCTTCAAGTCCGACAAGAAGCCCTCCGTCGACGAGGTCAAGAAGGTGCTCAACGACTACGTGTCCGAGCCACAAAAGCTGGGCTGTCCCTCCGCCCCCAAGAAGGCCATCCACCTGCTTGAGCAGCCCGACAGACCCCAGCCCCGTCTGGACCGAAACCGAGACAACGGCTACGCCGCATCTGTCGGCCGAGTCCGAGAGGACGCCGTGCTCGACTTCAAGTTCACCGTTCTATCCCACAACACTGTCATTGGTGCTGCCGGATCCGGTATTCTGATTGCCGAGACTCTGCTGAAGAAGAATCTCATCTAG* |
| *HOM6 (YALI0D01089g)* | *ATGTCCAAGGCTGTTAACATTGCTATTATCGGAACCGGCCTTGTCGGAAAGGCCTTCATCAACCAGCTTGCCGCCGTCAAATCCTCCATTGCCTACAATGTCGTTCTCATCGCTCGATCTTCTAAGACCCTCATCTCCAAGGACTTCAAGCCCCTCTCCCTGACCAACTGGGAGTCCGAGCTCAACTCCTCCCCCGTCGGAGCCATGTCTTTCACCGAGATCAACGACTTCCTCAAGAAGTCTCCTCTGCCCGTCATTCTGGTTGACAACACCTCCAACGAGGCCCTGGCCAACGAGTACCCTACTTTTGTCAACTCTGGTATCTCCATTGCCACCCCCAACAAGAAGGCCTTCTCCTCCGATCTTAAGACCTGGGAGGCCATCTTCGGCGGCGCTGAGAAGTCCGGCGCTCTTGTCTACCATGAGGCTACTGTCGGTGCCGGTCTGCCCGTCATCTCTACCCTCAATGACCTCATTGCCACCGGTGACGAGGTCGAaACtGTCGAGGGTATTGTTTCCGGAACTCTGTCTTACATCTTCAACGAGTTCTCCACCCTCTCTGGCTCTGACGTTAAGTTCTCCGACGTTGTCACCAAGGCCAAGCAGCTCGGATACACCGAGCCCGATCCCCGAGAGGATCTCAATGGTCTGGATGTCGCCCGAAAGGTCACCATTCTGGCTCGACTCTCTGGCTTCGACGTGGAGTCTCCCACTGCCTTCCCTGTTCAGTCCCTGATTCCCAAGCCCCTCGAGACTGCCTCTTCCGCCGATGAGTTCCTCCAGAAGCTCCCCGAGTATGACGCCGACCTTGCTAAGCTTCGAGACGAGGCTTTCGCCGAGAAGAAGGTTCTCCGATTCGTCGGTTCCATCAACAAGGGCACCGGCAAGGTCGAGGTTGGTATTCAGAAGTACGATGCCTCTCATCCCTTTGCCTCCCTCAAGGGCTCCGATAACATTATCGCCTTCAAGACCAAGCGATACCCCAACCCTCTGGTCATCCAGGGAGCTGGTGCTGGAGACGAGGTCACTGCTGCTGGTGTTCTTGCCGACGTTTTCAAGGCTGCCCAGCGACTTGCCAAATAG* |
| *THR1 (YALI0F13453g)* | *ATGTCTCGAAAGTTTGAAATTTCTGTGCCCGCGTCCTCGGCCAACATTGGTCCCGGTTTCGACGTGCTCGGCCTCGCTCTGGCCAAGTTCCTCGTGATCAACGTCGAAATCGACTCGTCCAAAACCTCGGACCGAAAGGACCCCAACAACTGCATTATCACCTACGAGGGCGAGGGAGCTGACGGCGTTCCTCTCGACAGTGACCACAACCTTGTCACCCGAGTGGCTCTCTACGTTCTGCGATGCAACGGCATTAGATCTTTCCCCTCGGGCACCTTTGTGCACGTCAACAACCCCATTCCTCTGGGTCGAGGTCTGGGCTCGTCTGGAGCGGCTGTGGTGGCTGGTGTCATGCTCGGAAACGAGGTCGGAAAGCTGGGCTTCTCCAAGCAGCGAATGCTCGACTATTGTCTCATGATCGAGCGACATCCCGACAACATCACCGCAGCCATGATGGGCGGCTTTGTGGGTTCTTACCTGCGGGAGCTGTCTGCTGCTGATCTCGAGCGAGTGGAGATCCCCCTTGCTGAGGTTCTCCCCGAGCCTGCCGGAGGCCGAGATACCGGCCTTGTACCCCCCGAGCCTCCTCTCAACATCGGACACCACATCAAGTACGACTGGTGCCGAGAAATCAAGGCCATTGTGGTGATTCCCAACTTTGAAGTGTCCACCGCCTCGGCCCGAGGAGTGCTCCCCACTGCCTACACTGCTTCAGATATGATCTTCAACCTGCAGCGACTGGCCGTTCTCACCACCGCCCTCACCCGATCGCCTCCCCAGGCCGACCTCATCTATCAGGCCATGCAGGACAAGGTCCACCAGCCCTACCGAAAGACCCTGATCCCCGGTCTGCCTGAGATTCTGGCCTCCGTTACCCCCAAGACTCACGAGGGTCTGCTGGGCATCTGTCTGTCTGGCGCTGGGCCCACCATTCTGGCTCTGGCCACCGACAACTTCGAGACAATCGCCAAGGAGATTGTGTCGCGATTCAACAAGGAGGGCATCGACTGCCGATGGGAGGTCCAGGAGTTGGCCTACGACGGTTCCAACGTGAAGCAGCTCTAA* |
| *THR4 (YALI0F23221g)* | *ATGACCACCTACTTTTCCACGCGATCATCCAATGAGCCCATTTCCTTCGAGGCTGCCGTCATGAAGGGTCTGGCCCCCGATGGAGGTCTTTACATTCCCACCTCCATCCCCAAGCTGCCCTCCGACTTCCTCACCAAGTGGGCCGATCTGAGCTTTGCCGAGTTGGCCTTCGAGATTCTCAGTCTGTACATTCCCGAGTCTGAGATCTCGCGAGCCGACCTGAAGGAGCTTGTCACCCGGTCCTACTCTACTTTCCGATCCGACGAGGTCACCCCCGTGGTTGAGCTCAGCAAGGAGAAGCAGCTTTATCTGCTGGAGCTATTCCACGGCCCCACCTACGCCTTCAAGGACGTGGCTCTGCAGTTTGTTGGCAACCTGTTTGAGTACTTCCTGACCCGAAAGAACGTTGGCAAGGAGGGCACCGACCGAGATACTCTTACCGTCGTGGGAGCCACCTCTGGTGACACCGGCTCCGCCGCCATCTACGGTCTGCGAGGCAAGAAGGACGTTTCTGTCTTCATTCTGTACCCCACCGGCCGAGTCTCCCCCATCCAGGAGGACCAGATGACGACTGTCTCTGACGCCAACGTGCACACTATCTCCGTTGCTGGTACCTTTGACGACTGCCAGGACATTGTTAAACAGGTCTTTGGTGATGCCGAGTTTAACGCCAAGCACCACGTCGGTGCCGTCAACTCTATCAACTGGGCCCGAATCCTCGCCCAGATCACCTACTACTTCCACTCCTTCTTCCAGGTCCAGAAGAAGTTCGGCACCTCCTCCGCCATCAAGTACTCTGTCCCCACTGGTAACTTTGGAGACATTCTCGCTGGCTTTTACGCCCGACGAATGGGTCTGCCCATCCAGGAGCTCACCATCGCCACTAACTCCAACGACATTCTCGACCGATTCCTCAAGACTGGCTCCTACTCGAAATCCGACGGAGCTTCTGCCGAGGTCCATGCCACCCTTTCCCCCGCCATGGACATCCTCGTCTCCTCCAACTTTGAGCGATTCCTTTGGTACGTTGCCCGAGAGAACGTTGCTTCTTCGGACGCTGAGGCTGGAGCCACCCTCAACAAGTGGATGCAGTCTCTCAAGACCGACGGTGTCATCACTGTCGACGCCAAGGTCCTTGGAGCCGCAAAGTCCGAGTTTTCCTCCGAGCGAGTCTCCGACGAGCAGACTCTCGAAACTATTAAGGATGTGTTTACCAACATTTCCAAGGGCTATATTTTGGACCCCCACTCCTCTGTTGGTGTCACTGCCGCTCTGCGAAAGCTTGAGGGAACCGACTCCGTGTACATTGCTCTGTCAACCGCTCACCCCGCCAAGTTCTCTGACGCCGTCGACCAGGCCCTCAAGGGCCTGGAGGGCTACAACTTTGAGCGAGATGTTCTCCCTCAGGAGTTCAAGGACTTTGCTAACAAGGACAAGAAGAAGCTGTTCAGCAAGGCCGACGTCAAGGAGGTCGAAAGCATCATTGAGGAGGAGCTGGCCAAGGAGAAGAAGCAGTAA* |
| *ILV1 (YALI0D02497g)* | *ATGTCCGAACCCGACTATCTGAAGCTCATCTTGAAGAGCCGCGTCTACGACGTGTGCAAGGAAACACCTGTGACATCTGCTCATGGTCTGAGCGAGAAGCTGGGCTGCAAAGTGCTGCTCAAGCGGGAAGATCTTCAGCCGGTTTTCTCGTTCAAGCTGCGAGGAGCCTACAACATGATTTCGCAGCTGAGTGACGAGGAAAAGTGGAAGGGAGTGATTGCGTGTAGCGCCGGTAACCATGCCCAAGGAGTCGCCTTTTCAGCCAACTATCTCAACATTCCAGCGACTATTGTCATGCCGTTGGCCACTCCTTCCATCAAGCACAGTAATGTTTCTAGACTAGGTGGCAAGGTGGTTTTGCACGGAGACGATTTTGATTCGGCCAAGGCCCACTGCAAGCAGCTGTGTGAGAAATATGGACTCACAGATATCCCTCCCTTTGATCACCCCCACGTGATTGCAGGCCAGGGAACTATTGGTATGGAGATTCTTCGTCAGGCGTCGGACAACCTGAAGGCCGTGTTTATCTGTGTTGGAGGCGGCGGTCTGATTGCCGGAGTAGGCGCTTACATCAAGCGGATCCAGCCCGATGTCAAAATCATTGCCGTGGAAACTTATGATGCATGTGCTCTGAAACAGAGTCTCATCAAGGGCGAACGGGTGACTCTGCCTGAAGTCGGTCTGTTTGCCGATGGAGCTGCTGTCAAGCTGTGTGGCGAGGAGACTTTCCGACTCTGTCGCAAGTACGTTGATGGAGTTGTGCTTGTGAACACGGACGAGATCTGCGCCGCTATCAAAGATGTATTTGAGGCCACTAGATCGGTGGTGGAGCCTGCTGGTGCTCTGTCGGTGGCTGGCCTGAAGAAGTACTGCTCCGACCCCTCGGCCATTTGGTGGTCACCTGAGTCCGATTCCGCAAAGGCCAATGGTATCCCCACTAACGTTGCCATCTCAGAAACCGACGAGTATCTGTCAATTCTCTCTGGAGCCAACATGAACTTTGACCGGCTTCGATTCGTGGCCGAACGAGCTATGCTTGGAGAAGGAACCGAAGTCTTCATGGTCGTCACCATCCCCGATATTCCCGGAGCGTTTGAAAAGCTGCACGAGATCATTCTCCCCAGAGCTGTCACCGAGTTCTCCTACAGAAAGAAGTCCACTGCTGAGAACGAAGACGCTAACATTTTTGTGTCTTTTTCAGTCAAAAACCGACAAGAGGAAATTGCAGACGTGCTGGAAAAGCTGCAAGCTGCCGGTATGAGCGGAGTCGACGTTTCAGACAACGAACTGGCAAAGACCCACGCTAGATATCTCGTGGGAGGCCAGCCAGACGTGCCTAATGAGAGACTGTTCCGGTTCGAGTTCCCTGAACGACCCAACGCGCTCAAAAACTTCCTCGGAGGTGTCCAGACAAAGTGGAATATCACCCTGTTCCACTACAGAAACAACGGCAGTGATATTGGAAAGATTCTGACAGCCTTGGACGTGCCGGAAAGCGACAATGAGGCGCTCAAGGAGTTTCTTGAGAAGCTCAAGTACCCCTTTGTGGAGGAGACAGACAATGTGGTGTACAAGCAGTTTATGAAGTAA* |
| *PDA1 (YALI0F20702g)* | *ATGTCCATCGCTGACGATGCCGACAAGAAATGCACAATCACGCTCAAGGAGGATTCTTACACCACCTACATGCTTGATTCTCCCCCTCCTCTCGAGTTCGAGATGACCAAGGGTGAGCTTCTGCAAATGTACAAGGACATGGTGACCGTCCGACGACTCGAGATGGCTGCTGATGCCCTCTACAAGGCCAAGAAGATCCGAGGTTTCTGCCATCTGTCTACTGGTCAGGAGGCTGTTGCCGTCGGTATCGAGAAGGCCATCGACCACGACGATTCTGTCATCACCGCCTACCGATGCCACGGTTTCGCCTACATGCGAGGTGCCTCTGTCCGAGCAATCATCGCCGAGCTGCTCGGAAAGCGAACCGGTGTCTCCTACGGTAAGGGTGGcTCCATGCACATGTTCACCGAGGGTTTCTACGGAGGAAACGGTATTGTCGGAGCCCAGGTCCCCGTCGGAGCTGGcCTCGCCTTCGCCCACAAGTACCTCGAGCAGACCGGAAAGGCCACCTTTGCCCTGTACGGTGACGGTGCTTCCAACCAGGGTCAGATCTTCGAGGCCTACAACATGGCCAAGCTCTGGGACCTCCCCTGCATCTTTGCATGCGAGAACAACAAGTACGGAATGGGTACCGCTGCTGCTCGATCCTCTGCCCTGACGCAGTACTACAAGCGAGGTCAGTACATTCCCGGcCTCAAGGTTAACGGAATGGACATTCTGTCCGTCTACCAGGGAGCCAAGTTCGCCAAGGAGTGGACCACACACGGCAAGGGTCCCCTCGTCATGGAGTTCGAGACtTACCGATACGGTGGTCACTCCATGTCCGATCCCGGAACCACCTACCGAACCCGAGAGGAGATCCAGTACATGCGATCCCACAACGATCCTATTTCTGGcCTCAAGGCCCACATCCTGGAGCTTAATTTCGCCACTGAGGACGAGCTTAAGTCTGTGGACAAGGCTGCTCGAGCTATGGTTGACAAGGAGGTTGCCCTTGCTGAGTCCGACCCTGCTCCTGAGGCTACTGCCAAGGTTCTGTTTGAGGATATCTACGTTCCCGGCACCGAGCCTCCTGTGATCCGAGGCCGAATCCCTTCCGAGGACTACTACTTTAAGAACTAA* |
| *PDB1 (YALI0E27005g)* | *ATGGCCTCAACCGAAGGCGGCGCCACTAACATGACTGTCAGAGACGCCCTCAACACCGCACTGCGAGAGGAGATGGACCGAAACGATAATGTTTTCATCATGGGTGAGGAGGTCGGCCAGTACAACGGTGCCTACAAGGTCACCAAGGGCCTTCTCGACAAGTTCGGCGAGAAGCGAGTGGTTGACACCCCTATCACCGAGATGGGTTTCGCCGGTGTTTGTGTCGGTGCCGCCCTGGCCGGcCTCACCCCCGTCTGCGAGTTCATGACCTGGAACTTCGCCATGCAGGCCATTGATCAGATCATCAATTCCGGTGCCAAGACCTACTACATGTCCGGAGGTACCCAGCAGTGCAATGTCACCTTCCGAGGTCCTAACGGTGCCGCCGCTGGTGTTGCTGCCCAACACTCTCAGGATTTCACCGGGTGGTACGGCCAGATTCCCGGcCTCAAGGTCGTCTCTCCCTACAGCTCTGAGGATGCCAAGGGTCTGCTCAAGGCCGCCATCCGAGAtCCCAACGTGACTGTTTTCCTCGAGAACGAGATCATGTACGGAGAGTCTTTCCCCATGTCTGAGGAGGCCATGTCCCCCGACTTCGTTCTGCCCCTTGGAAAGGCCAAGATTGAGCGAGAGGGTAAGGATATCACTCTTGTCGGTCACTCCCGAAACGTCGAGACtGCCCTCAAGGCCGCCGACCTCCTCAAGAAGCACCACAACGTCGATGCCGAGGTCATTAACCTGCGAACTGTCAAGCCTCTCGACACTGAGACtATTTTCAACTCCATCAAGAAGACTAACCGACTTGTCTCTGTCGAGGCTGGCTTCCCCGCCTTTGGCATGGGCTCCGAGCTCTGTGGTGTCGTCAACGACTCCTGGGCCTGGGATTACCTTGATGCCCCCATCCAGCGAGTTACCGGAGCTGAGGTTCCCACTCCTTACGCCATTGAGCTTGAGAACTTCGCCTTCCCCACACCCGAGATTGTTGTCAAGGCTGCCAAGGACGCCCTCTACATTGAGGAGTAG* |
| *LAT1 (YALI0D23683g)* | *ATGGCCGCCAAGTCCTGGCCTAGCCACACAGTCATCGACATGCCCGCCCTGTCCCCTACCATGACCCAGGGTAACATTGGCGCCTGGCAAAAGTCTGTCGGCGACGCTCTTGCTCCCGGCGAGGTTCTCGTCGAGATTGAGACTGACAAGGCCCAGATGGACTTTGAGTTCCAGGATGATGGCTACCTGGCCAAGATTCTGCTCGACGCCGGAGCCAAGGACATTGCCGTTGGCACCCCCATTGGTGTCTACGTCGAGGACGAGGCCGACGTGGCTGCCTTCAAGGACTTCACCATTGACGACGCCGGAGGAGTCCCCAAGCCTCCCAAGACCGAGGAGCAGAAGGAAGAGGAGGAATACGAGGCCGAGAAGGCCGAGAAGGCCGAGAAGGAGGCCGAGGCTTCCAAGGAGACTGCTTCTCCCGCCCCCTCTTCTCAGTCCTCTGCCCCTGCTGCCCCCACTCCCCCCTCTTCTCGAATCTTTGCTTCTCCCATGGCCAAGACCATTGCTCTGGAGAAGGGCATCAAGCTTAGCGAGATCAAGGGCTCCGGTCCCGGTGGTCGAATCATCAAGCGAGACGTCGAGAACTGGACCCCTCCCGCCGCTCCCGCCGCCAAGGCTGCCCCTGCCAAGGGCGCTGCCCCTGCTGCTGCCGCTGCTGCTGGATCTGCTTACACCGACATTCCTCTCACCAACATGCGAAAGACAATTGCTTCTCGACTGACCCAGTCCAAGAACACATCTCCCGACTACATTGTGTCTTCCACCGTGTCTGTGTCCAAGCTGCTCAAGCTGCGAGCTGCTCTCAACGCCTCCTCCGATGGTACCTACAAGCTGTCCATCAACGATCTGCTTGTCAAGGCCCTGGCCGTGGCCAACACCAAGGTCCCCCAGGTCAACTCCCAGTGGCTCGAGTCTGAGGGTGTTATTCGACAGTTCACCAACGTGGACGTTTCTGTCGCCGTTGCCACCCCCACTGGTCTGATCACCCCTGTGGTCAAGAACGCCAACCTCAAGGGTCTGGCGGAAATCTCCAAGGAGATTAAGGCTCTGGGCAAGAAGGCCAAGGACGGCAAGCTGGCTCCCGAAGAGTACCAGGGCGGTACCGTGACCATTTCCAACCTGGGCATGAACCACGCCGTGTCCTTCTTCACTGCCATCATCAACCCTCCCCAGGCCGCCATTCTCGCTGTCGGCACCACCGAGCGAAAGGCCATTGAGGACGTTGACTCCGAGGCCGGCTTTGTCTTTGACGACGTTGTTACTCTGACCACCTCTTTCGACCACCGAGTCGTCGATGGAGCTGTTGGAGGCGAGTGGGTCAAGGCTCTCAAGCAGGTGGTTGAAAACCCCATTGAGATGTTGTTGTAG* |
| *LPD1 (YALI0D20768g)* | *ATGAGCTCCGCAAACGAGGAGCTTGATGTCCTTGTTATCGGAGGTGGCCCCGGTGGATACGTCGCCGCCATCAAGGCCGCCCAGGCCGGCCTCAAGACCGGCTGTATCGAGAAGCGAGGCTCCCTCGGAGGAACCTGTCTCAACGTCGGATGTATCCCCTCTAAGTCTCTGCTCAACAACTCCCAGATGTACCACGCCATCAAAACCGACTCCGCCAACCGAGGAATCGAGGTgTCTGACGTCAAGATGAACATCGCCAAGCTGCAGGAGGCCAAGGAGACtTCCGTCAAGGGcCTCACCGGCGGTATCGAGATGCTGTTCAAGAAGAACAAGGTCAACTACTACAAGGGCGCCGGTTCTTTTGTGTCCGACTCCGAGGTCAAGGTCGACCCCATTGATGGCGGCGAGGCCGTCACCCTCAAGGCCAAGAACATCATCATTGCCACCGGCTCTGAGCCCACCCCCTTCCCCGGCATCACCATTGACGAGAAGAAGATTGTTTCCTCCACTGGTGCCCTTGCCCTCGAGGCCGTCCCCAAGAAGATGGTCATCATCGGAGGAGGTATCATTGGcCTCGAGATGGGCTCCGTCTGGTCCCGACTCGGCTCCGAGGTGACTGTTGTCGAGTTCCAGAACGCCATTGGCGCTGGTATGGACGACGAGATCGCCAAGGCCGCCCAGAAGATGCTCACCAAGCAGGGTATCAAGTTCAAGCTTGGCACCAAGGTGCTTTCCGGTGCCATTGAGGGCGACGGCGTCAAGGTCGAGGTCGAGAACGTCAAGAAGGGCGACAAGGAGACtCTTGATGCCGACGTTCTGCTCGTTGCCATTGGCCGACGACCCTACTCCGAGGGCTTGAACCTCGAGGCTGCCGGTGTCGAGAAGGACGACAAGGGCCGAATCATCATCGACCAGGAGTACCGAACCAACAAGTCCAACATCCGATGCATTGGTGACGTCACTTTCGGCCCCATGCTGGCCCACAAGGCCGAGGAGGAGGGAGTTGCTACCGCTGAGTACATTGCCACCGGTCACGGCCACGTTAACTACGCCGCCATCCCCTCTGTCATGTACACCCACCCTGAGGTTGCTTGGGTTGGACAGACTGAGCAGCAGGTCAAGGAGGCCGGCATCAAGTACAACGTCGGCAAGTTCCCCTTTGCCGCCAACTCTCGAGCCAAGACCAACCTCGACACCGAGGGTACCGTCAAGTTCATTGCTGACAAGGAGACTGACCGAATTCTCGGTATCCACATCATTGGCCCCAACGCCGGTGAGATGATCGCCGAGGGTGTTCTTGCCCTTGAGTACGGTGCTTCTTGCGAGGACATTGCTCGAACCTGCCACGCCCACCCCACTCTCTCCGAGGCTTTCAAGGAGGCCGCCATGGCCACTTACGACAAGGCCATCCACTTTTAA* |
| *PDX1 (YALI0B09845g)* | *ATGACCACCCCTCGCCTCTATCAGGCCAGCAACTTTGCCATGCCAGCCATGAGTCCCACCATGACCGAGGGAGGCATTGTGTCGTGGAAGGTCAAGGAGGGCGACGAGTTTTCAGCTGGTGATGTCATCCTTGAGATCGAAACCGACAAGGCCCAGATCGACGTCGAGGCTGCTGACGATGGAGTCATGGCCAAGATCTACAAGAAGGACGGTGACAAGGACATTCAGGTCGGAGACACCATTGCTGTGATTGCTGAGCCCGGAGATGACATCAAAACCATTGATATTCCTGCTCCTGTCGAGTCGGACGGCAAGCCTGCTCCCAAGGAGGAGGCCAAGGAGGAGGTCAAGGAGGCCCCTAAGGAGGAGGCTAAGGCCCCTGCCCCTAAGGCTCCTTCTACCCCCAAGGAGGCTCCCAAGACCGAATCGTCTTCTGCTCCCTCATCATCCGGCTACTCTGCCCCCGCTAACCCCGCACAGACCCTTCTCCCTTCGGTGTCCTCGCTTCTTGTGGCCAATGGCATCTCCAAGGAGGACGCCTTTGCCAAGATCAAGGCCACCGGTCCCCACGGCCGGCTTCTCAAGGGCGACATTCTCGCCTACCTCGGAAAGGTCCCTGAGGGCTCTCCTGGCGCTGTTGCCGACGAGATTAACAAGCGATCCCATCTCGACTTGTCCAACATCAAGCCCGCCAAGAAGAGCGATGCTGCTGCTCCCACTGGGGCTGCCAAGGCTGGAGATGCTGCTGCTCCTGCTACCAAGGCTGCTCCCGAGCCTGTCATCTTCACCTCGTTCCTCGATGTGGCCCACTTTGAGCCCGAGGACCAGCTCGAGCTGCAGCGAATCGTCAAGCAGGCCATCAAGCTGGCCAAATACGACGCCGTGGAGCTCGCAAAGCCCCGCCGATCCGCCAACGTGGACCCCGACTTTGAGGCTATCATCGGCCCCGCCAAGGGCACCAAGTTCTACGACGTGAACGTCATCTACCCCAAGTCCACAAAGCGGGCTCTCTCCAATGGGGCCGACCTGTATGACATTCTGTCGGACCGAAAGCCCCGAAAGGCTGCTGTCCAGGTGCCTTCCAACACTGTGCAGGTCGATGTAACAGTCAACGACAAGGTCCCCGGTGCCGAGAAGCGAGCCAAGCTTTTCTTGAACAGACTGGAGTATCATTTGACTGTGGAGGAGTAG* |

**Supplementary Table 4. Predicted enzyme locations for *S. cerevisiae* and *Y. lipolytica*.**

| ***S. cerevisiae*** | **Prediction program** | **CELLO** | | **MitoProt** | |
| --- | --- | --- | --- | --- | --- |
|  |  | **Location** | **Localization score** | **Location** | **Probability of exportation to mitochondria** |
|  | **THR1** | cytoplasm | cyto 1.422, mito 0.517 | cytoplasm | 0.0758 |
|  | **THR4** | cytoplasm | cyto 1.496, mito 0.899 | mitochondria | 0.8364 |
|  | **ILV1** | mitochondria | cyto 0.384, mito 3.104 | mitochondria | 0.9754 |
| ***Y. lipolytica*** | **Prediction program** | **CELLO** | | **MitoProt** | |
|  |  | **Location** | **Localization score** | **Location** | **Probability of exportation to mitochondria** |
|  | **THR1** | cytoplasm | cyto 1.542, mito 0.718 | cytoplasm | 0.0072 |
|  | **THR4** | cytoplasm | cyto 1.165, mito 0.674 | cytoplasm | 0.1128 |
|  | **ILV1** | cytoplasm | cyto 2.897, mito 0.353 | cytoplasm | 0.046 |

References

1) Yu CS, Cheng CW, Su WC, Chang KC, Huang SW, Hwang JK, and Lu CH*: CELLO2GO: A Web Server for Protein subCELlular LOcalization Prediction with Functional Gene Ontology Annotation. PLoS ONE 2014, 9(6): e99368.

2) Claros MG, Vincens P. Computational method to predict mitochondrially imported proteins and their targeting sequences. Eur. J. Biochem. 241, 770-786.
